# Determining the minimum important differences for field walking tests in adults with long–term conditions: a systematic review and meta-analysis

**DOI:** 10.1183/16000617.0198-2024

**Published:** 2025-05-28

**Authors:** Enya Daynes, Ruth E. Barker, Amy V. Jones, Jessica A. Walsh, Claire M. Nolan, William D-C. Man, Sally J. Singh, Neil J. Greening, Linzy Houchen-Wolloff, Rachael A. Evans

**Affiliations:** 1 Centre of Exercise and Rehabilitation Sciences, Leicester NIHR Biomedical Research Centre–Respiratory, University Hospitals of Leicester NHS Trust, Leicester, UK; 2 Department of Respiratory Sciences, University of Leicester, Leicester, UK; 3 Harefield Respiratory Research Group, Royal Brompton and Harefield Hospitals, Guy's and St Thomas’ NHS Foundation Trust, London, UK; 4 National Heart and Lung Institute, Imperial College, London, UK; 5 Wessex Academic Health Science Network, Southampton, UK; 6 Sydney School of Health Sciences, University of Sydney, Sydney, Australia; 7 College of Health Medicine and Life Sciences, Division of Physiotherapy, Brunel University London, London, UK; 8 Faculty of Life Sciences and Medicine, King's College London, London, UK

## Abstract

**Importance:**

The minimum important difference (MID) for field walking tests aims to improve interpretation of outcomes, but the volume and heterogeneity of MIDs for these tests is challenging. We aimed to determine the MID for the 6-min walk distance (6MWD), incremental shuttle walk test (ISWT) and endurance shuttle walk test (ESWT) in adults with long-term conditions.

**Methods:**

This systematic review included studies that generated a MID using an anchor-based approach in patients with long-term conditions for the 6MWD, ISWT or ESWT field walking tests. Studies were screened and data extracted by independent reviewers. Meta-analyses were performed using RevMan.

**Results:**

42 studies were included in the analyses, involving n=13 949 participants. Of these, 12 studies involving exercise as an intervention were included in the meta-analyses to produce MIDs, presented as mean (95% confidence interval). The MID for the 6MWD was 25 m (24–26 m) for respiratory conditions, 23 m (8–37 m) for cardiac conditions and 37 m (26–49 m) for neurological/musculoskeletal conditions. The MID for the ISWT was 48 m (39–57 m) for respiratory conditions and 70 m (55–85 m) for cardiac conditions. The MID for ESWT in COPD was 159 s (94–224 s). The pooled MID across conditions within exercise interventions was 26 m (22–40 m) for the 6MWD and 53 m (44–62 m) for the ISWT, with reasonable heterogeneity (I^2^=48% and I^2^=47%, respectively).

**Conclusion:**

We propose new MIDs for exercise interventions using anchor-based methodology in long‑term conditions for the 6MWD, ISWT and ESWT. These can be used internationally for meta‑analyses where studies have used different field walking tests, to optimise trial sample size calculations, and for clinical service benchmarking.

## Shareable abstract

**This review presents minimum important differences (MIDs) within long-term conditions. The pooled MID across conditions was 26** **m (22–30** **m) for the 6MWD and 53** **m (44–62** **m) for the ISWT with reasonable heterogeneity (I^2^=48% and I^2^=47%, respectively).** https://bit.ly/42ZKnQf

## Introduction

Approximately one in three older adults are living with a long-term condition globally [1]. Long-term conditions (or chronic conditions) are typically incurable and rely on management of symptoms using a combination of medical and non-medical therapies [2]. Patients with long-term conditions frequently have reduced exercise capacity and quality of life compared to healthy individuals, and present to healthcare settings with functional decline. Exercise-based interventions and therapies are primary treatments for a number of long-term conditions and are assessed through exercise testing. Field walking tests can be used as outcome measures and are also useful in prognostication of morbidity and mortality. They can also be used to prescribe and tailor exercise to the individual. Common field walking tests include the 6-min walk distance (6MWD), incremental shuttle walk test (ISWT) and endurance shuttle walk test (ESWT) [3].

A minimum important difference (MID) is the smallest difference in the outcome of interest that can indicate an improvement or deterioration of an intervention or condition, assessed by anchor-based methods or distribution methodologies such as standard error of measurement and effect size. Intervention quality and patient benefits using the MID would determine the level of change required for patients to perceive a clinical improvement [4]. Determining MIDs is important for clinical practice because they aid in determining the clinical impact and clinical relevance of the intervention [5]. These can be used as justification for policy, clinical practice and quality assurance. MIDs are important for research and in industry to determine the effectiveness of an intervention and to assist with sample size calculations for clinical trials [4].

Studies that have established a MID for the 6MWD, ISWT and ESWT use a variety of methodologies, and these provide different MIDs depending on the approach. MIDs for field walking tests have been calculated in a number of long-term conditions at varying stages of severity as well as in response to different interventions. It can be difficult to determine which MID is appropriate. The overarching aim of this systematic review is therefore to synthesise the literature determining the MIDs of the 6MWD, ISWT and ESWT using meta-analyses where appropriate in adults with long-term conditions to provide a range of MIDs for researchers and clinicians. This will allow them to select the most relevant MID for their disease area, intervention and by method of MID generation.

## Methods

The systematic review was prospectively registered through the international prospective register of systematic reviews (PROSPERO; reference CRD42020185565) and reported in accordance with the Preferred Reporting Items for Systematic Reviews and Meta-Analyses (PRISMA) guidance. The inclusion criteria for studies were participants aged ≥18 years old and generation of a MID using anchor-based methodology in a long-term condition. Studies that only used distribution methods (effect size) to generate a MID were excluded because theoretically this could include any intervention study using a field walking test in line with technical standards. Studies that provided both anchor- and distribution-based methods were used to enable direct comparison of generated MIDs through both methods in the same population. The outcomes were the 6MWD, ISWT distance and ESWT time [2]. There were no limitations on study design and systematic reviews were included where a new MID was generated.

### Search strategy

A search of PROSPERO was undertaken to identify existing and relevant systematic reviews; no similar reviews were identified. Five databases were searched from inception until September 2021 and updated in December 2024 (MEDLINE, Embase, CINAHL, EMCARE and Cochrane Central). Key search terms were structured around MID and outcome measures (6MWD, ISWT, ESWT) (supplementary material). Specific diseases were not part of the search terms so any disease not mentioned in the results has not had a MID evaluated for the field walking tests described in our search. Database searches were supplemented with forwards and backwards citation tracking with hand-searching of identified citations and duplicate citations removed. Titles and abstracts and subsequent full texts were screened by two independent reviewers.

### Data extraction and appraisal

Data were extracted independently by two reviewers and risk of bias was assessed using a modified COnsensus-based Standards for the selection of health Measurement INstruments (COSMIN). Studies were graded on a three-point scale (low, unclear or high risk of bias) for each domain and overall level of bias was determined. Training was provided to individuals performing the risk of bias assessment, the tool was piloted and discrepancies were discussed with an additional independent reviewer. Authors of the included studies were contacted for further data where needed. Studies for which the necessary data were not retrieved were excluded from the meta-analyses but were included in the narrative synthesis.

### Data analysis/synthesis

Extracted data were tabulated alongside the risk of bias and explored by exercise test, disease group and methods of determining MID. For each walking test, the population, intervention and methodology of generating an MID was described. MID estimates are presented as a range across studies. MIDs generated by anchor-based methodologies were investigated using a mean difference, random effects model meta‑analysis of the raw data and error of measurement, and analysed using RevMan. Where a confidence interval was not available, data were transformed using upper limit–lower limit/3.92, as per Cochrane recommendations. Data for which transformation was not possible (*i.e.* standard error of measurement not provided) were excluded from the meta-analysis. Where methods were similar (*e.g.* 0.5sd, effect size), MIDs derived from distribution-based methods were combined to generate an overall mean±sd. Sensitivity analyses were performed to include studies that had a low risk of bias where possible. Data were synthesised by intervention and categorised as respiratory, cardiac, neurological/musculoskeletal and other.

## Results

### Study characteristics and risk of bias

**FIGURE 1:**
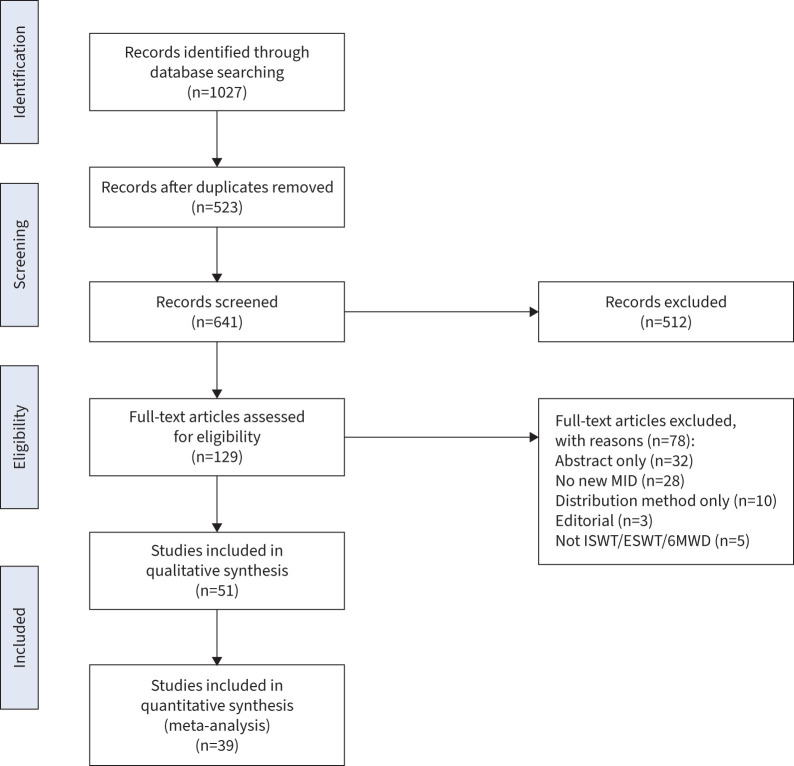
Consort diagram of included studies. 6MWD: 6-min walk distance; ESWT: endurance shuttle walk test; ISWT: incremental shuttle walk test; MID: minimum important difference.

Out of 641 studies, 51 studies exploring the MID using at least an anchor-based method were identified and included in the analysis (figure 1): 36 for the 6MWD [6–41], 10 for the ISWT [11, 42–50] and five for the ESWT [50–54]. The pooled sample size was n=15 718 with the study sample size ranging from n=22 to n=2404. The mean age range of participants in the included studies was 42–70 years and 58% were male (range 5–95%).

24 studies explored the MID in respiratory conditions, nine in cardiovascular disease, 12 in neurological/musculoskeletal conditions, two in pulmonary arterial hypertension, three in surgery and one in chronic kidney disease (table 1). Specific diseases were not part of the search terms so any disease not mentioned has not had a MID investigated for the field walking tests described in the relevant timeframe. In terms of risk of bias, there were n=25 studies that were at high risk, n=21 that were at moderate risk and n=5 that were at low risk of bias (table 1).

**TABLE 1:** Study characteristics for field walking tests

Study or subgroup	Subjects (n)	Disease	Method(s) of MID calculation	MID	Intervention	Risk of bias
**6MWD**						
**Respiratory**						
Chan (2015) [6]	641	Acute respiratory failure and ARDS	Anchor/linear regression: SF36 physical function domain	Modest improvement of 113 m	No intervention between 3 and 12 months	High
du Bois (2011) [7]	826	IPF	Distribution: including SEM and effect size	45 m, 95% CI 42–47 m	Interferon γ-1b 6MWT completed at baseline 24 weeks	Moderate
			Anchor: criterion reference approach of selected health events	24 m		
Granger (2015) [8]	56	Lung cancer	Anchor/ROC: European Organisation for Research and Treatment of Cancer Quality of Life Questionnaire	42 m, AUC 0.66, sensitivity 0.53, specificity 0.61	No intervention, 8–10 week follow-up	Moderate
			Distribution: SEM	22 m		
			Distribution: effect size	32 m		
Holland (2010) [10]	75	COPD	Anchor: global rating scale: “much worse”, “a little worse”, “about the same”, “a little better”, “much better”	24.5 m (20–61 m), AUC 0.83, sensitivity 0.85, specificity 0.67	Twice-weekly, 7-week PR with an individually prescribed exercise programme followed by self-management	Low
			Anchor: global rating scale: “much worse”, “a little worse”, “about the same”, “a little better”, “much better”	13.7%, AUC 0.78, sensitivity 0.7, specificity 0.78		
			Anchor: global rating scale (rated by clinician): “much worse”, “a little worse”, “about the same”, “a little better”, “much better”	23.5 m, AUC 0.8, sensitivity 0.83, specificity 0.75		
			Anchor: global rating scale (rated by clinician): “much worse”, “a little worse”, “about the same”, “a little better”, “much better”	13.7%, AUC 0.96, sensitivity 0.66, specificity 0.25		
			Distribution: effect size and SEM	SEM=25.5 m, moderate effect size=26.5 m		
Holland (2009) [9]	48	Diffuse parenchymal lung disease	Anchor: between individuals classified as “changed” or “unchanged”	30.5 m, AUC 0.89, sensitivity 0.73, specificity 0.94	8-week exercise training programme (some were participants in an RCT evaluating efficacy of exercise training in diffuse parenchymal lung disease)	Moderate
			Distribution: SEM was calculated using revised Jacobsen formula	33 m		
			Anchor (only subjects with IPF): between individuals classified as “changed” or “unchanged”	29 m, AUC 0.93		
			Distribution (IPF only): SEM was calculated using revised Jacobsen formula	34 m		
Lee (2014) [11]	37	Bronchiectasis	Anchor/ROC: patient–reported GRCQ 0=no change, 1=almost the same, 2=a little worse/better, 3=somewhat worse/better, 4=moderately worse/better, 5=a good deal worse/better, 6=a great deal worse/better and 7=a very great deal worse/better Scores of 0–1 were classed as no change, scores of 2–3 were small changes and scores of 4–7 were substantial changes	24.5 m, AUC 0.76, sensitivity 0.72, specificity 0.75	8-week exercise programme, twice weekly: 30 min lower limb endurance and 20 min circuit weight training	Moderate
			Anchor/ROC: clinician-reported GRCQ (as above)	22.5 m, AUC 0.81, sensitivity 0.75, specificity 0.61		
			Distribution: SEM	22.5 m		
			Distribution: effect size	20 m		
Nathan (2015) [12]	338	IPF	Distribution: included SEM and effect size	21.7 m	Placebo arm of the of the two phase 3 CAPACITY studies of pirfenidone	Moderate
			Anchor: criterion reference approach of hospitalisation or death	37 m, 95% CI 34–40 m		
Polkey (2013) [13]	1847	COPD	Anchor: FEV_1_	29.7±82.9 m	No intervention, 1–3 years	High
			Anchor: SGRQ change	29.7 m		
Puhan (2008) [15]	460	COPD	Distribution: SEM	35 m	6–10 weeks PR	High
			Distribution: 0.5sd	29 m		
			Distribution: empirical rule effect size	42 m		
Puhan (2011) [14]	1218	COPD	Anchor/linear regression: SGRQ total	24.6 m, 95% CI 23.4–25.7 m	Respiratory rehabilitation (nine included studies, varying lengths)	High
			Anchor/linear regression: SGRQ Impacts domain	18.9 m, 95% CI 18.1–20.1 m		
			Anchor/linear regression: SGRQ Activity domain	24.2 m, 95% CI 23.4–25.4 m		
			Distribution: SEM	30.6 m		
Redelmeier (1997) [16]	112	COPD	Anchor: mean difference in 6MWD between patients rating themselves as “about the same” and mean difference in 6MWD between patients rating themselves as “a little better”	54 m, 95% CI 37–71 m	No intervention, distances between patients compared but not stated	Moderate
Swigris (2010) [17]	123	IPF	Baseline to 6 months: distribution: effect size (change in 6MWD/sd of baseline 6MWD)	41.3 m	Bosentan treatment, measured at 6 and 12 months	Moderate
Zampogna (2021) [30]	37	Asthma	Anchor: MRC dyspnoea scale	26 m, 95% CI 0–117 m	Inpatient, daily PR for 3 weeks	Moderate
**Surgery**						
Antonescu (2014) [18]	119	Colorectal surgery	Anchor: linear regression models used to estimate the change in mean scores between adjacent levels of the anchor on each domain of health, activity levels and 6MWD	Between-group estimate 19 m, 95% CI 9–30 m Within-person estimate 14 m, 95% CI 9–18 m	Surgery	High
Murao (2024) [33]	243	Allogenic HSCT	Anchor: patient-reported outcome Quality of Life Questionnaire-q2 MID -1	−37.5 m change post stem cell transplant is an important decrease	Physical therapy five times per week from before the start of conditioning regimens of HSCT until discharge from hospital	Low
Sheraz (2022) [34]	89	CABG	Anchor: GRCS 7-point Likert scale	195 m; AUC 0.651, 95% CI 0.510–0.792	Cardiac rehabilitation for 7 days	High
			Distribution: SEM	13.03 m		
Yanagisawa (2024) [35]	97	Colorectal cancer surgery	Anchor: EQ5D change	−60 m	Surgery+postoperative rehabilitation (twice daily on weekdays and once on Saturdays, for a total of 40–60 min·day^−1^ for 7 days)	High
**PAH**						
Gabler (2012) [32]	2404	PAH	Meta-regression to determine threshold of significant reduction in clinical events	41.8 m	No intervention, follow-up at 12 weeks	High
Mathai (2012) [19]	405	PAH	Anchor: physical component summary score	38.6 m	Either tadalafil 2.5, 10, 20 or 40 mg orally once daily or placebo for 16 weeks	Moderate
			Distribution: effect size, standardised response mean, SEM, 0.5sd of the baseline measure Triangulated to generate a clinically and statistically relevant measure of change	33 m, 95% CI 15–50 m		
**Cardiac**						
Gremeaux (2011) [28]	81	Coronary artery disease	Anchor: self-assessment of clinical change (2 points) slightly better or meaningful change	25 m, AUC 0.78, sensitivity 0.55, specificity 0.92	8-week cardiac rehabilitation (educational intervention based on patient's risk factors and personalised training using the results of a stress test performed on a treadmill using the Bruce modified protocol). Sessions lasted 1.5 h, 3 days per week over 8 weeks: 2×30 min aerobic exercise and 20 min circuit weight training.	Moderate
			Anchor: clinician's assessment of clinical change (2 points) slightly better or meaningful change	25 m, AUC 0.78, sensitivity 0.7, specificity 0.78		
			Distribution: SEM (patient's assessment)	23 m		
			Distribution: SEM (clinician's assessment)	36 m		
Igarashi (2024) [36]	35	Inpatients with subacute cardiac disease	Anchor: patient-determined GRCS	ROC 27.5 m, 95% CI 22–42.5 m	No intervention	Moderate
			Anchor: professional-determined GRCS	ROC 32.5 m, 95% CI 29.5–37.5 m		
			0.5sd	18.1 m		
Khan (2023) [37]	680	CHF	Anchor: patient global assessment tool 7-point Likert scale	14 m, 95% CI 5–23 m	Intravenous ferric carboxymaltose	High
Shoemaker (2013) [20]	22	CHF	Anchor: GCRQ 5-point Likert scale, compared improved *versus* not improved	30.1 m, 95% CI 20.8–39.4 m	8-week observational study; participants instructed to maintain their usual clinical routine	Moderate
Täger (2014) Cohort 1 [21]	461	CHF	One-SEM-based approach (MID=sd 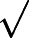 (1−r))	35 m	No intervention with 6-month follow-up	High
Täger (2014) Cohort 2 [21]	512	CHF	One-SEM-based approach (MID=sd 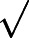 (1−r))	37 m	No intervention with 6-month follow-up	High
**Non-cardiorespiratory conditions**						
Benaim (2019) [22]	437	Lower limb, neck or back pain	Anchor: GRCQ scale −3 to +3 (much worse to much better, respectively)	Lower limb 75 m, neck/back 60 m	4–6 weeks inpatient therapy sessions, 5 days·week^−1^; sessions included physiotherapy, physical reconditioning, occupational therapy, vocational therapy and cognitive-behavioural therapy	High
			Distribution: SEM	Lower limb 58 m, neck/back 55 m		
			Opinion-based Delphi	83 m		
Claeys (2024) [38]	85	Pompe disease	Anchor: PROMIS >2-point change	4.93±7.05 m	No intervention	High
			Anchor: SGIC	4.45±6.84 m		
			Anchor: forced vital capacity 3%	4.85±6.99 m		
Forrest (2014) [23]	249	Incomplete spinal cord injury	Correlation and regression method	0.11 m·s^−1^, AUC 0.85, 0.81, 0.81 (39.6 m converted to ms)	Standardised locomotor training therapy programme (described elsewhere)	Moderate
Fulk (2018) [24]	265	Cerebral vascular accident	Anchor: Stroke Impact Scale	65 m, AUC 0.59, sensitivity 0.68, specificity 0.5	Early locomotor training (2 months post stroke) *versus* late locomotor training (6 months post stroke), 90 mins three times per week for 12–16 weeks for both interventions	High
			Anchor: Modified Rankin Scale (measure of stroke disability)	71 m, AUC 0.66, sensitivity 0.7, specificity 0.58		
Gardner (2018) [29]	180	Peripheral artery disease	Anchor: SF6 physical function scale	32 m	Supervised *versus* home-based *versus* light training 3 days per week for 3 months	Moderate
			Distribution	32 m		
Kaleth (2016) [25]	187	Fibromyalgia	Anchor/linear regression: Fibromyalgia Impact Questionnaire Total	156 m	RCT: all participants received an individualised exercise programme and two supervised exercise sessions; intervention group received six exercise-based motivational interviewing phone calls; control group received six health education phone calls	High
			Anchor: SF36-PF	167 m		
Kwok (2013) [26]	73	Moderately frail older adults	Distribution: SEM	12.9 m	12-week outpatient rehabilitation: one supervised session and at least two home sessions per week, including balance, strengthening, aerobic, stretching	High
			Anchor: GRCQ −7 to +7 (a great deal worse to a great deal better, respectively)	17.8 m, AUC 0.7, sensitivity 56.7, specificity 83.3		
Lika (2024) [39]	55	Pompe disease	Anchor: SF36 categorised as better, same or worse	0.35%	No intervention	High
			Distribution: 0.5sd	4.53 m	No intervention	
King (2022) [40]	278	Knee osteoarthritis	Anchor: 8-point Likert scale of perceived improvement	74.36 m	Knee arthroplasty	High
Oosterveer (2022) [41]	118	Multiple sclerosis	Anchor: 3-point Likert scale	19.7 m	No intervention	Moderate
Spina (2019) [27]	42	Chronic inflammatory demyelinating polyradiculoneuropathy	Anchor: GRCQ 5-point scale from significantly worse to significantly better	20.26 m, 95% CI 4.07–30.7 m	High-dose *i.v.* immunoglobulin (0.4 g·kg^−1^·day^−1^) or *i.v.* methylprednisolone (500 mg·day^−1^) for 5 days, 2 months duration	High
			Distribution: minimal detectable change (1.96sd×sem)	20 m		
			Distribution: effect size (small effect size used as estimate)	19.96 m		
Zeitlberger (2021) [31]	49	Lumbar degenerative disc disease	Anchor: VAS-back	81 m	6 weeks postoperatively (spinal surgery), performed using a digital version of the 6MWD	High
			Anchor: Core Outcome Measure Index-back	92 m		
			Anchor: Zurich Claudication Questionnaire	99 m		
			MDC (95% CI)	114 m		
**ISWT**						
**Respiratory**						
Cartlidge (2018) [42]	57	Bronchiectasis	Anchor/ROC: SGRQ ≥5% improvement	AUC 0.79, sensitivity 0.92, specificity 0.50	12 months nebulised gentamycin	Moderate
Evans (2019) [43]	613	COPD	Distribution: 0.5sd of the change in ISWT distance	36.1 m	7-week course of PR	Low
			Anchor: GRCQ 1–5 (much better to much worse)	35 m; AUC 0.66, sensitivity 0.7, specificity 0.67 66.7 m, 95% CI 52.19–81.21 m		
Lee (2014) [11]	37	Bronchiectasis	Anchor/ROC: GRCQ patient perspective 0=no change, 1=almost the same, 2=a little worse/better, 3=somewhat worse/better, 4=moderately worse/better, 5=a good deal worse/better, 6=a great deal worse/better, 7=a very great deal worse/better [12]. Scores of 0–1 were classed as no change, scores of 2–3 were small changes and scores of 4–7 were substantial changes.	35 m, AUC 0.88, sensitivity 0.88, specificity 0.83	8-week exercise programme, twice weekly: 30 min lower limb endurance and 20 min circuit weight training	High
			Anchor/ROC: GRCQ clinician perspective (as above)	35 m, AUC 0.8, sensitivity 0.69, specificity 0.83		
			Distribution: sem	37 m		
			Distribution: effect size	32 m		
Nolan (2018) [44]	72	IPF	Distribution: 0.5sd	35 m	8-week twice-weekly outpatient PR	High
			Distribution: sem	31 m		
			Anchor: GRCQ	46 m, 95% CI 18–74 m		
Singh (2008) [45]	372	COPD	Anchor: GRCQ 5-point Likert scale (1=much better to 5=much worse)	47.5 m (38.6–56.4 m)	7-week outpatient PR	Moderate
Walsh (2020) [46]	119	Bronchiectasis	Anchor: CRQ	CRQ-D, CRQ-F, CRQ-T mean (range) of MCID estimates: 58 m (95% CI 45–70 m), AUC CRQ‑D: 0.70, CRQ‑F: 0.70, CRQ‑T: 0.68; sensitivity CRQ‑D: 60.0, CRQ‑F: 67.7, CRQ‑T: 69.6; specificity CRQ‑D: 68.9, CRQ‑F: 63.2, CRQ‑T: 61.9	8-week twice-weekly outpatient PR	High
**Cardiac**						
Hanson (2019) [47]	52	Coronary heart disease	Anchor: 95% limit cut-off point	Improvement: Change 1st pre and post test: 92 m; Change 2nd pre and post test: 82 m	8-week cardiac rehabilitation	Moderate
			Anchor/ROC: GRCQ (improved, same, worse)	Improvement: Change 1st pre and post test: 85 m; Change 2nd pre and post test: 85 m		
Houchen-Wolloff (2015) [48]	220	Post myocardial infarction; post percutaneous coronary intervention; post CABG (23.2%)	Anchor: GRCQ 5-point Likert scale	70 m, 95% CI 51.5–88.5 m, or a 25% improvement when assessed at a population level, or seven whole shuttles	Cardiac rehabilitation; 6 weeks, one supervised session per week lasting 90 min, circuit based	Low
			Distribution: sd method	36.65 m		
Sheraz (2022) [34]	89	CABG	Anchor: GRCS 7-point Likert scale	42.5 m, AUC 0.849, 95% CI 0.759–0.939	Cardiac rehabilitation for 7 days	High
			Distribution: SEM	3.99 m		
**Non-cardiorespiratory conditions**						
Wilkinson (2019) [49]	26	Non-dialysis chronic kidney disease	Anchor: SF36	45 m, 95% CI 3–66 m	Exercise rehabilitation; three times per week for 12 weeks, moderate to vigorous exercise	Moderate
			Distribution: sd (0.5sd of the change score)	29 m		
			Distribution: effect size (change in scores corresponding to a small effect size (0.2) (0.2=the mean change score))	6 m		
**ESWT**						
**Respiratory**						
Altenburg (2015) [50]	55	COPD	6MWD (25 m), *W*_peak_ (4 W) and CRQ (10 points total score)	*W*_peak_=198.9 s 95% CI 163.7–234.1, 81.2%, 163.6 m Anchor: CRQ=186.4 s, 95% CI 147.4–225.4, 75.9%, 153.7 m Anchor: 6MWD=199.1 s, 95% CI 153.3–245, 82.2% (metres NA because not highly correlated)	12-week rehabilitation with or without nocturnal noninvasive intermittent positive pressure ventilation	Moderate
			Distribution: 0.5sd	145 s, 137 m and 61.4%		
Borel (2014) [51]	276	COPD	Distribution: sem	61 s or 82 m	8-week multicentre, placebo-controlled, double-blind, randomised, parallel-group study investigating whether adding fluticasone/salmeterol fixed-dose combination to open label tiotropium further improved walking capacity in comparison to tiotropium alone	Moderate
			Anchor: GRCQ 7-point Likert scale	56.3 s, 95% CI 48.9–63.6, 69.6 m		
Hill (2019) [52]	78	COPD	Distribution: standardised response mean calculated as 0.5sd of the change in performance on the ESWT following training	156 s or 188 m	8–10 week walking training programme, individually tailored, ground based, 2–3 supervised sessions per week. Initial training intensity prescribed to equivalent of 80% of average walking speed achieved from better of two 6MWTs. Total exercise time=30 min at start of programme and increased by 5 min after every sixth session to a maximum of 45 min.	Moderate
			Anchor: participants rating their walking ability after training as a “little better” from those who did not	70 s, 95% CI −91–231 s or 80 m		
			SEM: square root of the mean square of the random error term derived using a repeated ANOVA	227 s and 317 m		
Pepin (2011) [53]	PR: 132, Bronchodilation: 69	COPD	Distribution (PR cohort): 0.5sd	186 s	PR cohort: 7 weeks PR three times per week Bronchodilation cohort: bronchodilation Unable to calculate PR cohort anchor–based MID with confidence	High
			Distribution (bronchodilation cohort): 0.5sd	70 s		
			Anchor (bronchodilation cohort): GRCQ 7-point Likert scale (−3=large deterioration to +3=large improvement)	65 s, 95% CI 45–85 s		
Zatloukal (2019) [54]	531	COPD	Anchor/ROC: ISWT	207 s, AUC 0.77, sensitivity 0.702, specificity 0.699	6-week twice-weekly outpatient PR	Low
			Anchor: GRCQ 7-point Likert scale (−3=large deterioration to +3=large improvement)	279.2 s, 95% CI 244.9–313.5 s		
			Distribution: 0.5sd	173.7 s		

6MWD: 6-min walking distance; 6MWT: 6-min walking test; ARDS: acute respiratory distress syndrome; AUC: area under the curve; CABG: coronary artery bypass graft; CHF: chronic heart failure; CRQ: Chronic Respiratory Questionnaire (D: Dyspnoea, F: Fatigue, T: Total domains); ESWT: endurance shuttle walking test; FEV_1_: forced expiratory volume in 1 s; GRCQ: Global Rating of Change Questionnaire; HSCT: haematopoietic stem cell transplant; IPF: idiopathic pulmonary fibrosis; ISWT: incremental shuttle walking test; MCID: minimum clinically important difference; MDC: minimal detectable change; MID: minimum important difference; MRC: Medical Research Council; PAH: pulmonary arterial hypertension; PR: pulmonary rehabilitation; PROMIS: Patient-Reported Outcomes Measurement Information System; RCT: randomised controlled trial; ROC: receiver operating characteristic; se: standard error; SEM: standard error of measurement; SF36: Short Form 36; SGIC: Subject Global Impression of Change; SGRQ: Saint George's Respiratory Questionnaire; VAS: visual analogue scale.

#### Intervention description for each walking test

The interventions in the included studies were exercise-based interventions, pharmacotherapy or no intervention where longitudinal studies investigated the outcome of interest, *e.g.* mortality over time.

For the 6MWD, 15 studies involved an exercise or rehabilitation intervention [8–11, 14, 20, 22, 24–26, 28–30, 32, 33], five involved pharmacological interventions [7, 12, 19, 27, 36], five studies following surgery [18, 31–34] and eight longitudinal studies involved no intervention [6, 9, 13, 15–17, 21, 32]. The exercise/rehabilitation interventions were pulmonary rehabilitation (n=5) [9–11, 14, 15], cardiac rehabilitation (n=3) [19, 27, 33] or rehabilitation for neurological or musculoskeletal conditions (n=6) [22–25, 28, 41]. For the ISWT, nine studies involved an exercise or rehabilitation intervention and one pharmacological intervention. All included studies investigating the ESWT (n=5) involved a pulmonary rehabilitation intervention ranging from 6 to 12 weeks [50–54]. One of these studies involved pulmonary rehabilitation and nocturnal noninvasive ventilation [50].

#### Methodologies

There was a range of different methodologies used to generate an MID. A variety of anchors were used to define a MID for the 6MWD, including the Global Rating of Change Questionnaire (GRCQ), patient‑reported questionnaires and one that used forced expiratory volume in 1 s. For the ISWT, seven studies used the GRCQ for an anchor; three used patient-reported outcome measures and one a walking test (6MWD). For the ESWT, four MIDs were generated using the GRCQ as an anchor and two used an exercise test (6MWD, cardiopulmonary exercise test). The distribution methods were standard error of measurement (6MWD n=20, ISWT n=5, ESWT n=7) and effect size (6MWD n=8, ISWT n=4).

### Description of MIDs among walking tests for all interventions and longitudinal studies

#### 6MWD

36 studies explored the MID for the 6MWD with a pooled sample size of n=12 984 (table 1) [6–41]. One study was excluded from the meta-analysis because the MID was in m·s^−1^, for which we were unable to generate a measurement error [23]. One study (with three generated MIDs) was excluded from the synthesis because a digital (non–standardised) version of the 6MWD was used [31]. There were 71 MIDs that ranged from −60 m to +195 m.

13 studies (n=5818) recruited patients with a respiratory condition [6–17, 30]. Two studies (n=2809) explored MID in patients with pulmonary arterial hypertension with a MID range of 33–42 m [19, 32]. Five studies (n=1791) explored the MID in cardiovascular disease with a range of 14–37 m [19, 20, 27, 36, 37]. Twelve studies (n=2018) explored an MID in neurological and musculoskeletal conditions, with a range of 13–83 m [22–27, 29, 31, 38–41]. Four studies (n=548) reported the MID in patients following surgery and reported an MID of between −60 m and +195 m [18, 33–35].

There were 22 distribution-determined MIDs for the 6MWD [6–10, 12, 14, 15, 17, 19, 21, 22, 26–29, 34, 36, 39, 50]. Using distribution methods, the MID range was 12–58 m. For respiratory disease (n=6) it was 14–45 m, for cardiac disease (n=3) it was 23–37 m and for neurological/musculoskeletal disease (n=4) it was 5–58 m. The range of MID using an effect size measurement was 20–42 m (n=6).

#### ISWT

Nine studies explored an MID for the ISWT with a pooled sample size of n=1657 (table 1) [11, 42–49]. The calculated MID ranged from 4 m to 92 m. Five studies (n=1270) calculated an MID for patients with a respiratory condition, which ranged from 31 m to 59 m. Three studies (n=361) explored the MID in patients with cardiovascular disease, with the MID ranging from 4 m to 92 m [34, 47, 50]. One study (n=26) explored the MID in patients with renal disease, calculated as 45 m (95% CI 3–66 m) [49].

There were nine MIDs produced from six studies using a distribution method alongside an anchor method for the ISWT [11, 34, 44, 45, 49, 50]. The range of MIDs was 5–37 m. Five studies used a distribution standard error of measurement with a range of 4–37 m. Three studies used an effect size, with the MID ranging 6–36 m.

#### ESWT

Five studies explored the MID for the ESWT with a pooled sample size of n=1141 (table 1) [50–54]. The MID ranged from 56 s to 279 s. All included studies reported MID in patients with COPD.

Seven MIDs were calculated from five studies for the ESWT using a distribution method, all using a standard error of measurement. The MID ranged from 61 s to 277 s (supplementary table S1) [50–54].

### Meta-analyses to describe the MID of walking tests for exercise interventions

Due to the volume of generated MIDs and heterogeneity of interventions, the data synthesis reports exercise-based interventions only and synthesises the MID using a meta-analysis for anchor-based approaches and a pooled mean±sd for distribution methods for all included conditions.

#### 6MWD

**FIGURE 2:**
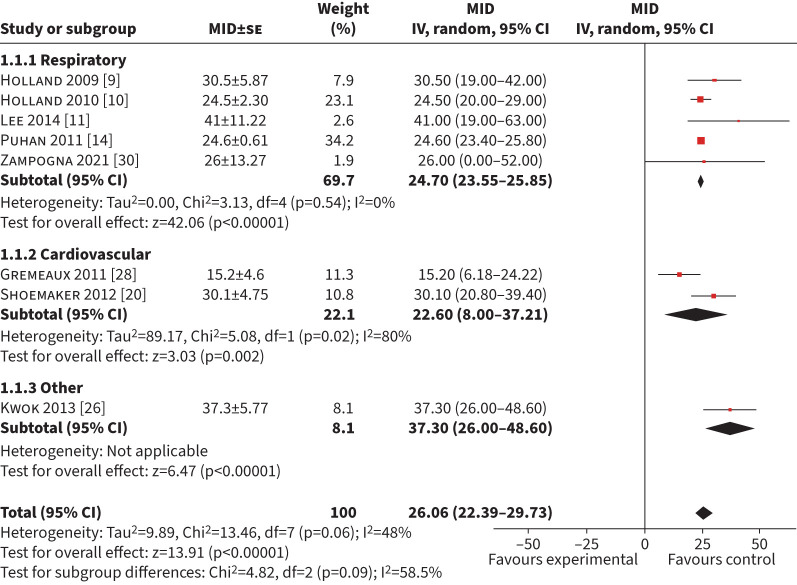
Forest plot of the 6-min walk distance minimum important difference (MID) calculated in exercise-based interventions. IV: inverse weighted; se: standard error.

A total of 15 studies (n=3485) explored exercise interventions using an anchor-based approach [9–11, 14, 15, 20, 22–26, 28–30, 34]. Eight studies (n=1633) were included in the meta-analysis for a MID of the 6MWD using an anchor-based approach [9–11, 14, 20, 26, 28, 30]. Nine studies were excluded because an error of measurement for the MID could not be calculated or obtained from the authors [15, 22, 24, 29–34]. The mean (95% CI) MID was 26 m (22–30 m) with an I^2^ of 48% (figure 2). Of the eight included studies, five (n=1378) included respiratory conditions with a mean (95% CI) MID of 25 m (24–26 m) (I^2^=0%). Two studies (n=103) in cardiac disease had a mean (95% CI) MID of 23 m (8–37 m) (I^2^=80%) [20, 28] and one study (n=73) included musculoskeletal/neurological conditions with a mean (95% CI) MID of 37 m (26–48 m) [26]. Six studies (n=336) used a GRCQ as an anchor with a pooled mean (95% CI) MID of 27 m (23–31 m) (I^2^=45) (supplementary table S1) [9–11, 20, 26, 28]. Sensitivity analysis on studies with a low risk of bias was not performed because only one study had a low risk of bias with a mean (95% CI) MID of 24 m (20–29 m) [10].

#### ISWT

**FIGURE 3:**
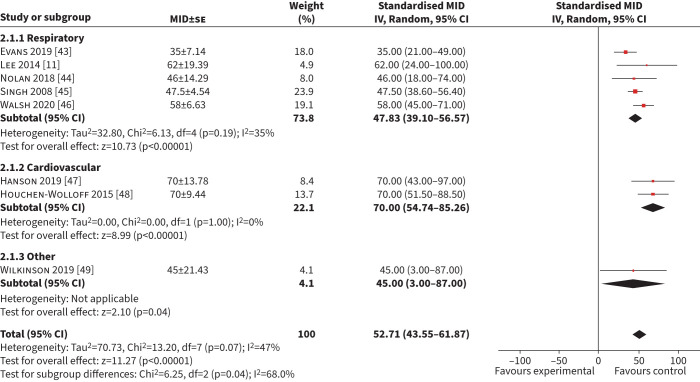
Forest plot of the incremental shuttle walk test minimum important difference (MID) calculated in exercise-based interventions. IV: inverse weighted; se: standard error.

Eight studies (n=1511) were included in the meta-analysis of MID for the ISWT using an anchor-based approach [10, 33–39.] The combined mean (95% CI) MID was 53 m (44–62 m) (I^2^=47%) (figure 3). Five studies (n=1270) included respiratory patients [11, 43–46], with a mean (95% CI) MID of 48 m (39–57 m) (I^2^=35%). Two studies (n=272) included patients with cardiac disease with a mean (95% CI) MID of 70 m (55–85 m) (I^2^=0%) [47, 48]. Six studies (n=1366) used a GRCQ as an anchor, with a mean (95% CI) MID of 52 m (18–86 m) (I^2^=57%) (supplementary table S1) [11, 43–45, 47, 48]. Two studies (n=145) used a health–related quality of life measure with a mean (95% CI) MID of 57 m (44–69 m) (I^2^=0%) [46, 49]. Two studies (n=833) had a low risk of bias and the mean (95% CI) MID was 52 m (18–86 m) (I^2^=89%) [43, 48].

#### ESWT

**FIGURE 4:**
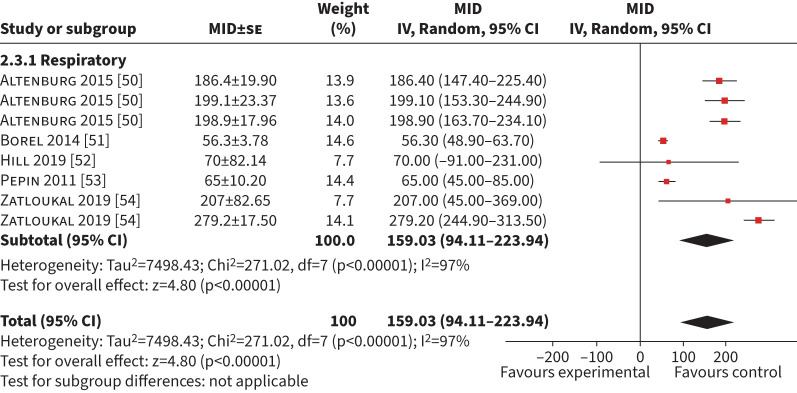
Forest plot of the endurance shuttle walk test minimum important difference (MID) calculated in exercise-based interventions. IV: inverse weighted; se: standard error.

Five studies generating eight MID (n=940) using anchor-based methods were included in the synthesis [50–54]. The mean (95% CI) MID was 159 s (94–224 s) (I^2^= 97%) (figure 4). Four studies (n=950) used a GRCQ, producing a mean (95% CI) MID of 123 s (36–209 s) (I^2^=98%) (supplementary table S1) [52–54]. Two studies (n=586) used an exercise test as an anchor (ISWT, 6MWD and peak work rate (W_peak_)) with a combined mean (95% CI) MID of 199 s (172–227 s) (I^2^=0%) [50, 54]. Sensitivity analysis on studies with a low risk of bias was not possible because only one study (n=531) had a low risk of bias (MID 276 s, 243–310 s) [54].

## Discussion

### Main findings

We report the results of a novel comprehensive systematic review of MID in long-term conditions for field walking tests involving 51 studies and n=15 718 participants. We report new mean (95% CI) MIDs of 26 m (22–30 m) for the 6MWD and 53 m (44–62 m) for the ISWT for all long-term conditions across exercise intervention studies, anchors and interventions. We report mean (95% CI) MIDs for exercise interventions in specific long-term conditions for the 6MWD (respiratory conditions: 25 m (24–26 m); cardiac conditions: 23 m (8–37 m); neurological/musculoskeletal conditions: 37 m (26–49 m)), the ISWT (respiratory conditions: 48 m (39–57 m); cardiac conditions: 70 m (55–85 m)) and for ESWT in respiratory disease (159 s (94–224 s). In addition, we provide reference tables (supplementary table S1 and figure S1) for different long-term conditions with methodology- and intervention-specific MIDs that will be useful for sample size determination. These findings are important to guide clinicians and researchers to the appropriate MID available, allowing consideration of the particular disease, intervention and method of generating the MID. Where there is an absence of disease-specific data, we recommend the use of the overall pooled MID as a best estimate. MIDs are often utilised for large interventional studies and in service evaluation and improvement projects such as the UK National Respiratory Audit Programme. Our results can provide valuable context on the impact of services on patient improvements [55]. We also highlight an evidence gap for MIDs for the ISWT and ESWT for neurological/musculoskeletal conditions and for the ESWT in cardiac conditions.

We report the MID using anchor methodology. Anchor-based methods and distribution-based methods yielded similar MIDs for exercise interventions. Many of the 6MWD studies used a 2-point “no change” *versus* “change” scale whereas studies for the ISWT used a 5- or 7-point Likert scale, which may affect the calculated MIDs. Two studies in this review used receiver operating characteristic curves in a similar population to determine an MID for the 6MWD and ISWT using pulmonary rehabilitation as the intervention, which yielded similar MIDs (25 m and 35 m, respectively) [10, 43]. These values enable comparison of interventions where either the 6MWD or ISWT have been used. This is valuable for service benchmarking and for meta-analysis of research studies.

It is important to consider the method, intervention and population groups when selecting an appropriate MID because each variable can affect the calculated MID. It has been suggested that the MID should distinguish between the methods by stating how it was calculated (*i.e.* MID-S for statistical methods and MID-P for patient determined) and this may assist in unravelling the complexities of MID [3]. Additionally, some may use a patient-determined GRCQ and some use a clinician-determined GRCQ. This can affect the generated MID and may explain the wide confidence intervals, though the variation of MID appears highest in those with cardiovascular conditions. Additionally, other anchors were used, such as mortality, validated health-related quality of life measures or another exercise test. The differences in the anchor will affect the generated MID and therefore create variability in the literature that our review synthesises. There is a lack of data for ESWT in conditions outside of respiratory diseases, despite it being sensitive to exercise interventions [3]. This review synthesised data for exercise-based interventions only because there were sufficient studies conducted whereas there were minimal studies for other interventions such as surgery and pharmacological interventions.

### Strengths and limitations

This is the first systematic review to synthesise the MID of field walking tests for long-term conditions, using different methodology, anchors and interventions. This is vital given the plethora of MIDs available in the literature (40 studies). Data are provided to support researchers and clinicians in selecting appropriate MIDs for their population and intervention. However, the data are limited to the available literature, which is biased towards respiratory disease. In the ESWT, this is limited to COPD only. It is common for studies to include patients in a stable state, and those with significant comorbidities are often excluded. Therefore, while there are a large number of different populations included, the application of these MIDs in adults with multiple long-term conditions or in a less stable state needs to be explored [5]. Studies that included distributions methods only (*i.e.* effect size) in the absence of an anchor-based method were excluded: it would be challenging to systematically identify all studies with distribution‑based MID because any trial could potentially generate an effect size but may not be adequately powered.

There are other factors that potentially affect the MIDs. Exercise-based interventions are generally 6–12 weeks duration, and therefore it is unlikely that disease decline will influence the MID whereas it might in studies of a longer duration. Baseline disease severity may influence the MID and therefore study population characteristics should be considered in the interpretation and use of the MID. Ideally the study sample would be representative of the entire disease population, but rehabilitation studies, for example, are biased towards more severe disease and patients with greater functional impairments. In order to compare MIDs across populations, the functional impairment would need to be comparable across groups [56]. The healthy survivor effect, whereby participants with more severe disease may drop out or not survive to the end of the study period, will skew the calculated MID [57].

This review focused on anchor-based methods and compared the statistical methods of the same population where it was reported. An MID to determine clinical improvement cannot signify perceived improvement without the participant's/patient's perspective on improvement. Distribution-based methods describe an improvement over and above the intrinsic variability of the measurement [58]. Therefore, when selecting and applying MIDs, researchers/clinicians need to consider the specific aim of the study/intervention, the population and disease progression in the context of the study duration. In study interpretation, the MID can signify if a treatment should be implemented or not and can help shape policy by demonstrating clinical benefit. This work demonstrates the multitude of MIDs available and the importance of selecting the most appropriate MID for the needs of the study or clinical service evaluation.

## Conclusion

This systematic review and meta-analysis has demonstrated the large volume of available MIDs described for field walking tests among different long-term conditions, and synthesised the results within exercise-based studies. The most appropriate MID should be selected based on disease and methodology. The mean (95% CI) MID for the 6MWD was 25 m (24–26 m) for respiratory conditions, 23 m (8–37 m) for cardiac conditions and 37 m (26–49 m) for neurological/musculoskeletal conditions. For the ISWT, the MID was 48 m (39–57 m) for respiratory conditions and 70 m (55–85 m) for cardiac disease. For the ESWT in respiratory disease, the MID was 159 s (94–224 s). The pooled MID across available conditions was 26 m (22–30 m) for the 6MWD, 53 m (44–62 m) for the ISWT and 159 s (94–224 s) for the ESWT, with a moderate to high heterogeneity (I^2^=48%, I^2^=47%, I^2^=97%, respectively).

The disease population, intervention and statistical method can affect the generated MID, and researchers and clinicians should consider these differences when selecting the most appropriate MID for their cohort.

**Points for clinical practice**

This review presents minimum important differences (MIDs) for field walking tests in long-term conditions. The pooled MID across conditions was 26 m (22–30 m) for the 6MWD and 53 m (44–62 m) for the ISWT with reasonable heterogeneity (I^2^=48%, I^2^=47%, respectively). This provides a useful indicator in clinical practice to compare services in order to understand clinical improvements.

## Supplementary material

10.1183/16000617.0198-2024.Supp1 **Please note:** supplementary material is not edited by the Editorial Office, and is uploaded as it has been supplied by the author.
Supplementary material ERR-0198-2024.SUPPLEMENT

## Data Availability

This is a secondary analysis containing group data only. Extracted data can be shared upon a written request to the corresponding author.

## References

[1] Hajat C, Stein E. The global burden of multiple chronic conditions: a narrative review. Prev Med Rep 2018; 12: 284–293. doi: 10.1016/j.pmedr.2018.10.008 30406006 PMC6214883

[2] Department of Health. Long Term Conditions Compendium of Information. Third Edition. Available from: https://assets.publishing.service.gov.uk/media/5a7c638340f0b62aff6c154e/dh_134486.pdf Date last updated: 30 May 2012.

[3] Holland AE, Spruit MA, Troosters T, et al. An official European Respiratory Society/American Thoracic Society technical standard: field walking tests in chronic respiratory disease. Eur Respir J 2014; 44: 1428–1446. doi: 10.1183/09031936.00150314 25359355

[4] Houchen-Wolloff L, Evans RA. Unravelling the mystery of the 'minimum important difference’ using practical outcome measures in chronic respiratory disease. Chron Respir Dis 2019; 16: 1479973118816491. doi:10.1177/1479973118816491 30789024 PMC6323555

[5] Guyatt GH, Osoba D, Wu AW, et al. Methods to explain the clinical significance of health status measures. Mayo Clin Proc 2002; 77: 371–383. doi:10.4065/77.4.371 11936935

[6] Chan KS, Pfoh ER, Denehy L, et al. Construct validity and minimal important difference of 6-minute walk distance in survivors of acute respiratory failure. Chest 2015; 147: 1316–1326. doi:10.1378/chest.14-1808 25742048 PMC4420183

[7] du Bois RM, Weycker D, Albera C, et al. Six-minute-walk test in idiopathic pulmonary fibrosis: test validation and minimal clinically important difference. Am J Respir Crit Care Med 2011; 183: 1231–1237. doi:10.1164/rccm.201007-1179OC 21131468

[8] Granger CL, Holland AE, Gordon IR, et al. Minimal important difference of the 6-minute walk distance in lung cancer. Chron Respir Dis 2015; 12: 146–154. doi:10.1177/1479972315575715 25749346

[9] Holland AE, Hill CJ, Conron M, et al. Small changes in six-minute walk distance are important in diffuse parenchymal lung disease. Respir Med 2009; 103: 1430–1435. doi:10.1016/j.rmed.2009.04.024 19477109

[10] Holland A, Hill C, Rasekaba T, et al. Updating the minimal important difference for six-minute walk distance in patients with chronic obstructive pulmonary disease. Arch Phys Med Rehabil 2010; 91: 221–225. doi:10.1016/j.apmr.2009.10.017 20159125

[11] Lee AL, Hill CJ, Cecins N, et al. Minimal important difference in field walking tests in non-cystic fibrosis bronchiectasis following exercise training. Respir Med 2014; 108: 1303–1309. doi:10.1016/j.rmed.2014.07.006 25087836

[12] Nathan SD, du Bois RM, Albera C, et al. Validation of test performance characteristics and minimal clinically important difference of the 6-minute walk test in patients with idiopathic pulmonary fibrosis. Respir Med 2015; 109: 914–922. doi:10.1016/j.rmed.2015.04.008 25956020

[13] Polkey MI, Spruit MA, Edwards LD, et al. Six-minute-walk test in chronic obstructive pulmonary disease: minimal clinically important difference for death or hospitalization. Am J Respir Crit Care Med 2013; 187: 382–386. doi:10.1164/rccm.201209-1596OC 23262518

[14] Puhan MA, Chandra D, Mosenifar Z, et al. The minimal important difference of exercise tests in severe COPD. Eur Respir J 2011; 37: 784–790. doi:10.1183/09031936.00063810 20693247 PMC5516638

[15] Puhan MA, Mador MJ, Held U, et al. Interpretation of treatment changes in 6-minute walk distance in patients with COPD. Eur Respir J 2008; 32: 637–643. doi:10.1183/09031936.00140507 18550610

[16] Redelmeier DA, Bayoumi AM, Goldstein RS, et al. Interpreting small differences in functional status: the six minute walk test in chronic lung disease patients. Am J Respir Crit Care Med 1997; 155: 1278–1282. doi:10.1164/ajrccm.155.4.9105067 9105067

[17] Swigris JJ, Wamboldt FS, Behr J, et al. The 6 min walk in idiopathic pulmonary fibrosis: longitudinal changes and minimum important difference. Thorax 2010; 65: 173–177. doi:10.1136/thx.2009.113498 19996335 PMC3144486

[18] Antonescu I, Scott S, Tran TT, et al. Measuring postoperative recovery: what are clinically meaningful differences? Surgery 2014; 156: 319–327. doi:10.1016/j.surg.2014.03.005 24947644

[19] Mathai SC, Puhan MA, Lam D, et al. The minimal important difference in the 6-minute walk test for patients with pulmonary arterial hypertension. Am J Respir Crit Care Med 2012; 186: 428–433. doi:10.1164/rccm.201203-0480OC 22723290 PMC3443803

[20] Shoemaker MJ, Curtis AB, Vangsnes E, et al. Clinically meaningful change estimates for the six-minute walk test and daily activity in individuals with chronic heart failure. Cardiopulm Phys Ther J 2013; 24: 21–29. doi:10.1097/01823246-201324030-00004 23997688 PMC3751711

[21] Täger T, Hanholz W, Cebola R, et al. Minimal important difference for 6-minute walk test distances among patients with chronic heart failure. Int J Cardiol 2014; 176: 94–98. doi:10.1016/j.ijcard.2014.06.035 25049008

[22] Benaim C, Blaser S, Léger B, et al. “Minimal clinically important difference” estimates of 6 commonly-used performance tests in patients with chronic musculoskeletal pain completing a work-related multidisciplinary rehabilitation program. BMC Musculoskelet Disord 2019; 20: 16. doi:10.1186/s12891-018-2382-2 30611242 PMC6320580

[23] Forrest GF, Hutchinson K, Lorenz DJ, et al. Are the 10 meter and 6 min walk tests redundant in patients with spinal cord injury? PLoS One 2014; 9: e94108. doi:10.1371/journal.pone.0094108 24788068 PMC4006773

[24] Fulk G, He Y. Minimal clinically important difference of the 6-minute walk test in people with stroke. J Neurol Phys Ther 2018; 42: 235–240. doi:10.1097/NPT.0000000000000236 30138230

[25] Kaleth A, Slaven J, Ang D. Determining the minimal clinically important difference for 6-minute walk distance in fibromyalgia. Am J Phys Med Rehabil 2016; 95: 738–745. doi:10.1097/PHM.0000000000000485 27003201 PMC5831244

[26] Kwok BC, Pua YH, Mamun K, et al. The minimal clinically important difference of six minute walk in Asian older adults. BMC Geriatr 2013; 13: 23. doi:10.1186/1471-2318-13-23 23510291 PMC3599457

[27] Spina E, Topa A, Iodice R, et al. Six-minute walk test is reliable and sensitive in detecting response to therapy in CIDP. J Neurol 2019; 266: 860–865. doi:10.1007/s00415-019-09207-1 30721354

[28] Gremeaux V, Troisgros O, Benaim S, et al. Determining the minimal clinically important difference for the six-minute walk test and the 200-meter fast walk test during cardiac rehabilitation program in coronary artery disease patients after acute coronary syndrome. Arch Phys Med Rehabil 2011; 92: 611–619 doi:10.1016/j.apmr.2010.11.023 21440707

[29] Gardner A, Montgomery P, Wang M. Minimal clinically important differences is treadmill, 6-minute walk, and patient-based outcomes following supervised and home-based exercise in peripheral artery disease. Vasc Med 2018; 23: 349–357. doi:10.1177/1358863X18762599 29671381 PMC6062461

[30] Zampogna E, Ambrosino N, Centis R, et al. Minimal clinically important difference of the 6-min walking test in patients with asthma. Int J Tuberc Lung Dis 2021; 25: 215–221. doi:10.5588/ijtld.20.0928 33688810

[31] Zeitlberger AM, Sosnova M, Ziga M, et al. Assessment of the minimum clinically important difference in the smartphone-based 6-minute walking test after surgery for lumbar degenerative disc disease. Spine (Phila Pa 1976) 2021; 46: E959–E965. doi:10.1097/BRS.0000000000003991 34042414

[32] Gabler NB, French B, Strom BL, et al. Validation of 6-minute walk distance as a surrogate end point in pulmonary arterial hypertension trials. Circulation 2012; 126: 349–356. 22696079 10.1161/CIRCULATIONAHA.112.105890 PMC4237273

[33] Murao M, Kondo T, Hamada R, et al. Minimal important difference of the 6-minute walk test after allogenic hematopoietic stem cell transplantation. Disabil Rehabil 2024; 46: 3449–3456. doi:10.1080/09638288.2023.2246013 37574839

[34] Sheraz S, Ayub H, Ferraro FV, et al. Clinically meaningful change in 6 min walking test and the incremental shuttle walking test following coronary artery bypass graft surgery. Int J Environ Res Public Health 2022; 19: 14270. doi:10.3390/ijerph192114270 36361150 PMC9655553

[35] Yanagisawa T, Tatematsu N, Horiuchi M, et al. Responsiveness and minimal clinically important difference of the 6-minute walk distance in patients undergoing colorectal cancer surgery. Support Care Cancer 2024; 32: 382. doi:10.1007/s00520-024-08596-y 38789578

[36] Igarashi T, Miyata K, Tamura S, et al. Minimal clinically important difference in 6-minute walk distance estimated by multiple methods in inpatients with subacute cardiovascular disease. Physiother Theory Pract 2024; 40: 1981–1989. doi:10.1080/09593985.2023.2232014 37395670

[37] Khan MS, Anker SD, Friede T, et al. Minimal clinically important differences in 6-minute walk test in patients with HFrEF and iron deficiency. J Card Fail 2023; 29: 760–770. doi:10.1016/j.cardfail.2022.10.423 36332897

[38] Claeys KG, Kushlaf H, Raza S, et al. Minimal clinically important differences in six-minute walking distance in late-onset Pompe disease. Orphanet J Rare Dis 2024; 19: 154. doi:10.1186/s13023-024-03156-3 38605392 PMC11008008

[39] Lika A, Andrinopoulou ER, van der Beek NAME, et al. Establishing how much improvement in lung function and distance walked is clinically important for adult patients with Pompe disease. Eur J Neurol 2024; 31: e16223. doi: 10.1111/ene.16223 38375606 PMC11235921

[40] King LK, Hawker GA, Stanaitis I, et al. Minimal clinically important difference for improvement in six-minute walk test in persons with knee osteoarthritis after total knee arthroplasty. BMC Musculoskelet Disord 2022; 23: 307. doi:10.1186/s12891-022-05262-4 35361173 PMC8969367

[41] Oosterveer DM, Van Den Berg C, Volker G, et al. Determining the minimal important change of the 6-minute walking test in multiple sclerosis patients using a predictive modelling anchor-based method. Mult Scler Relat Disord 2022; 57: 103438. doi:10.1016/j.msard.2021.103438 34871859

[42] Cartlidge MK, Smith MP, Bedi P, et al. Validation of the incremental shuttle walk test as a clinical end point in bronchiectasis. Chest 2018; 154: 1321–1329. doi:10.1016/j.chest.2018.09.019 30300653

[43] Evans RA, Singh SJ. Minimum important difference of the incremental shuttle walk test distance in patients with COPD. Thorax 2019; 74: 994–995. doi: 10.1136/thoraxjnl-2018-212725 31147399

[44] Nolan CM, Delogu V, Maddocks M, et al. Validity, responsiveness and minimum clinically important difference of the incremental shuttle walk in idiopathic pulmonary fibrosis: a prospective study. Thorax 2018; 73: 680–682. doi:10.1136/thoraxjnl-2017-210589 28883090

[45] Singh SJ, Jones PW, Evans R, et al. Minimum clinically important improvement for the incremental shuttle walking test. Thorax 2008; 63: 775–777. doi:10.1136/thx.2007.081208 18390634

[46] Walsh J, Barker R, Wynne S, et al. The minimal clinically important difference of the incremental shuttle walk test in bronchiectasis: a prospective cohort study. Ann Am Thorac Soc 2020; 17: 375–378. doi:10.1513/AnnalsATS.201907-563RL 31682480 PMC7044696

[47] Hanson LC, Taylor NF, McBurney H. Interpreting meaningful change in the distance walked in the 10-metre ISWT in cardiac rehabilitation. Heart Lung Circ 2019; 28: 1804–1811. doi:10.1016/j.hlc.2018.11.014 30591397

[48] Houchen-Wolloff L, Boyce S, Singh S. The minimum clinically important improvement in the incremental shuttle walk test following cardiac rehabilitation. Eur J Prev Cardiol 2015; 22: 972–978. doi:10.1177/2047487314540840 24958737

[49] Wilkinson T, Watson E, Xenophontos S, et al. The ‘minimum clinically important difference’ in frequently reported objective physical function tests after a 12-week renal rehabilitation exercise intervention in nondialysis chronic kidney disease. Am J Phys Med Rehabil 2019; 98: 431–437. doi:10.1097/PHM.0000000000001080 30362979

[50] Altenburg WA, Duiverman ML, ten Hacken NHT, et al. Changes in the endurance shuttle walk test in COPD patients with chronic respiratory failure after pulmonary rehabilitation: the minimal important difference obtained with anchor- and distribution-based method. Respir Res 2015; 16: 27. doi:10.1186/s12931-015-0182-x 25849109 PMC4336738

[51] Borel B, Pepin V, Mahler DA, et al. Prospective validation of the endurance shuttle walking test in the context of bronchodilation in COPD. Eur Respir J 2014; 44: 1166–1176. doi:10.1183/09031936.00024314 25186261

[52] Hill K, Ng C, Wootton SL, et al. The minimal detectable difference for endurance shuttle walk test performance in people with COPD on completion of a program of high-intensity ground-based walking. Respir Med 2019; 146: 18–22. doi:10.1016/j.rmed.2018.11.013 30665513

[53] Pepin V, Laviolette L, Brouillard C, et al. Significance of changes in endurance shuttle walking performance. Thorax 2011; 66: 115–120. doi:10.1136/thx.2010.146159 21148135

[54] Zatloukal J, Ward S, Houchen-Wolloff L, et al. The minimal important difference for the endurance shuttle walk test in individuals with chronic obstructive pulmonary disease following a course of pulmonary rehabilitation. Chron Respir Dis 2019; 16: 1479973119853828. 10.1177/1479973119853828.

[55] Royal College of Physicians. National Asthma and COPD Audit Programme. www.nrap.org.uk Date last accessed: 4 August 2022.

[56] Schunemann H, Guyatt G. Commentary- Goodby M(C)ID! Hello MID, Where do you come from? Health Serv Res 2005; 40: 593–597. doi:10.1111/j.1475-6773.2005.0k375.x 15762909 PMC1361157

[57] Jones P, Beeh K, Chapman K, et al. Minimal clinically important differences in pharmacological trials. Am J Respir Crit Care Med 2014; 189: 250–255. doi:10.1164/rccm.201310-1863PP 24383418

[58] Troosters T. How important is a minimal difference? Eur Respir J 2011; 37: 755. doi:10.1183/09031936.00156410 21454895

