# Supplementary material for: Determining the minimum important differences for field walking tests in adults with long–term conditions: a systematic review and meta-analysis
Source: Eur Respir Rev. 2025 May 28;34(176):240198. doi: 10.1183/16000617.0198-2024 (PMC12117381; doi:10.1183/16000617.0198-2024)
Supplement: Supplementary file 1 [file ERR-0198-2024.SUPPLEMENT.pdf]

Online supplement

S1 Search Strategy

Strategy 790356

#	Database	Search term	Results
1	Medline	("6 minute walk* test" OR 6MWT).ti,ab	4614
2	Medline	("six minute walk* test").ti,ab	1715
3	Medline	(incremental shuttle walk* test).ti,ab	349
4	Medline	(iswt).ti,ab	199
5	Medline	(Endurance Shuttle Walk* Test).ti,ab	153
6	Medline	WALK TEST/	1195
7	Medline	(1 OR 2 OR 3 OR 4 OR 5 OR 6)	6609
8	Medline	"MINIMAL CLINICALLY IMPORTANT DIFFERENCE"/	200
9	Medline	(minimal clinically important difference).ti,ab	1676
10	Medline	(minimal clinically significant difference).ti,ab	1239
11	Medline	(minimal important difference).ti,ab	3134
12	Medline	(minimal clinically important improvement).ti,ab	901
13	Medline	(minim*4 ADJ5 difference).ti,ab	6731
14	Medline	(MCID).ti,ab	1201
15	Medline	(8 OR 9 OR 10 OR 11 OR 12 OR 13 OR 14)	8791
16	Medline	(7 AND 15)	133
17	EMBASE	("6 minute walk* test" OR 6MWT).ti,ab	11188
18	EMBASE	("six minute walk* test").ti,ab	3944

19	EMBASE	(incremental shuttle walk* test).ti,ab	600
20	EMBASE	(iswt).ti,ab	520
21	EMBASE	(Endurance Shuttle Walk* Test).ti,ab	198
22	EMBASE	exp "WALK TEST"/	10422
23	EMBASE	(17 OR 18 OR 19 OR 20 OR 21 OR 22)	19219
24	EMBASE	"MINIMAL CLINICALLY IMPORTANT DIFFERENCE"/	1422
25	EMBASE	"MINIMAL CLINICALLY IMPORTANT DIFFERENCES"/	1395
26	EMBASE	"MINIMAL DETECTABLE CHANGE"/	49
27	EMBASE	(minimal clinically important difference).ti,ab	2093
28	EMBASE	(minimal clinically significant difference).ti,ab	48
29	EMBASE	(minimal important difference).ti,ab	629
30	EMBASE	(minimal clinically important improvement).ti,ab	148
31	EMBASE	(minim*4 ADJ5 difference).ti,ab	9888
32	EMBASE	(MCID).ti,ab	2431
33	EMBASE	(24 OR 25 OR 26 OR 27 OR 28 OR 29 OR 30 OR 31 OR 32)	11090
34	EMBASE	(23 AND 33)	348
35	CINAHL	("6 minute walk* test" OR 6MWT).ti,ab	1214
36	CINAHL	("six minute walk* test").ti,ab	820
37	CINAHL	(incremental shuttle walk* test).ti,ab	145
38	CINAHL	(iswt).ti,ab	77
39	CINAHL	(Endurance Shuttle Walk* Test).ti,ab	59

40	CINAHL	(35 OR 36 OR 37 OR 38 OR 39)	1861
41	CINAHL	(minimal clinically important difference).ti,ab	1030
42	CINAHL	(minimal clinically significant difference).ti,ab	679
43	CINAHL	(minimal important difference).ti,ab	1669
44	CINAHL	(minimal clinically important improvement).ti,ab	545
45	CINAHL	(minim*4 ADJ5 difference).ti,ab	3466
46	CINAHL	(MCID).ti,ab	605
47	CINAHL	(41 OR 42 OR 43 OR 44 OR 45 OR 46)	4265
48	CINAHL	(40 AND 47)	54
49	EMCARE	("6 minute walk* test" OR 6MWT).ti,ab	2452
50	EMCARE	("six minute walk* test").ti,ab	839
51	EMCARE	(incremental shuttle walk* test).ti,ab	132
52	EMCARE	(iswt).ti,ab	91
53	EMCARE	(Endurance Shuttle Walk* Test).ti,ab	35
54	EMCARE	exp "WALK TEST"/	2835
55	EMCARE	(49 OR 50 OR 51 OR 52 OR 53 OR 54)	4808
56	EMCARE	"MINIMAL CLINICALLY IMPORTANT DIFFERENCE"/	495
57	EMCARE	"MINIMAL CLINICALLY IMPORTANT DIFFERENCES"/	475
58	EMCARE	"MINIMAL DETECTABLE CHANGE"/	36
59	EMCARE	(minimal clinically important difference).ti,ab	749
60	EMCARE	(minimal clinically significant difference).ti,ab	13

61	EMCARE	(minimal important difference).ti,ab	153
62	EMCARE	(minimal clinically important improvement).ti,ab	50
63	EMCARE	(minim*4 ADJ5 difference).ti,ab	2601
64	EMCARE	(MCID).ti,ab	586
65	EMCARE	(56 OR 57 OR 58 OR 59 OR 60 OR 61 OR 62 OR 63 OR 64)	2954
66	EMCARE	(55 AND 65)	83

Figure S1 Calculated Minimal Important Differences for the 6 minute walking distance for all included studies

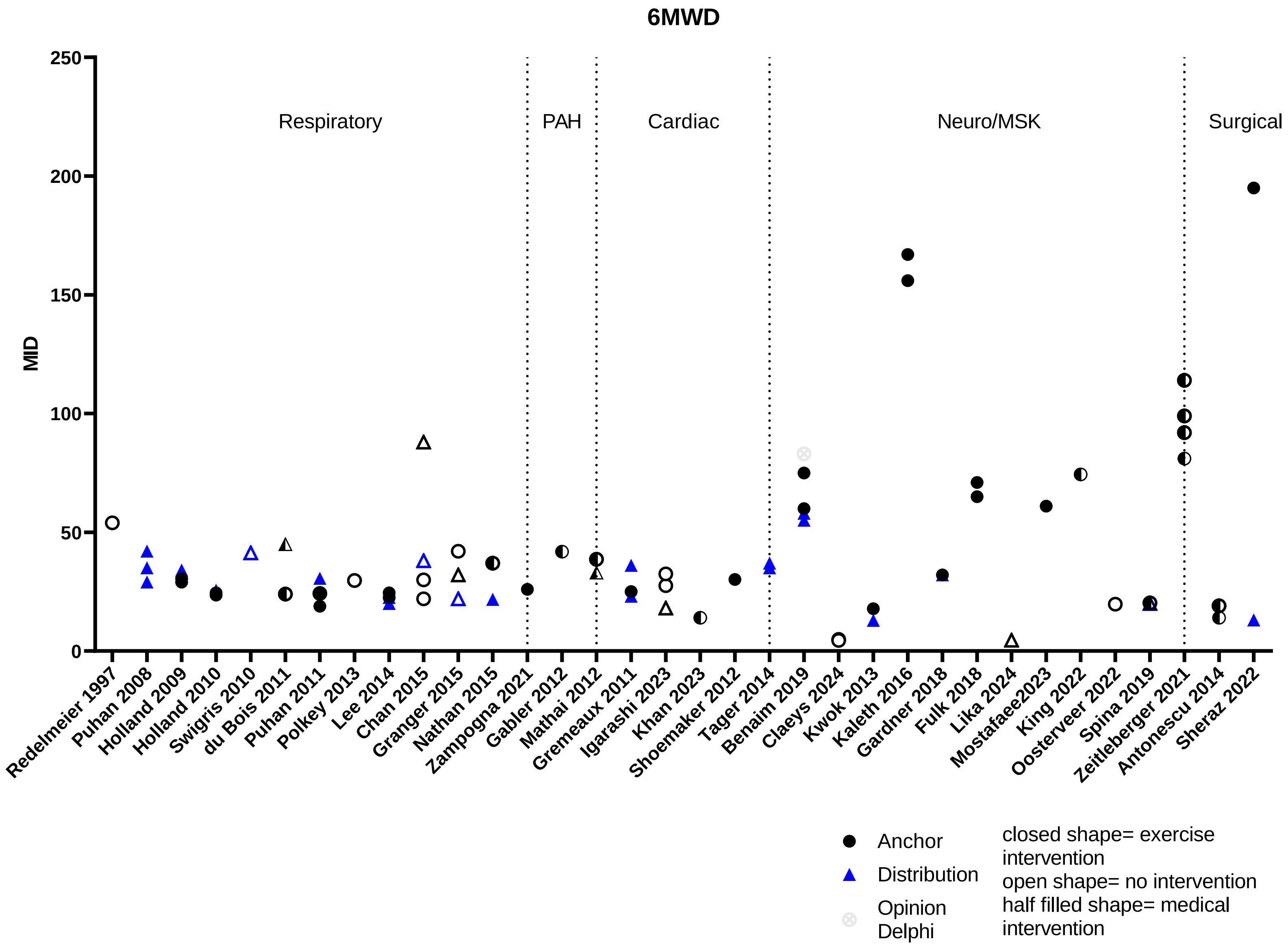
6MWD
MID
250
200
150
100
50
0
Respiratory
PAH
Cardiac
Neuro/MSK
Surgical
Redelmeier 1997
Puhan 2008
Holland 2009
Holland 2010
Swigris 2010
du Bois 2011
Puhan 2011
Polkey 2013
Lee 2014
Chan 2015
Granger 2015
Nathan 2015
Zampogna 2021
Gabler 2012
Mathai 2012
Gremeaux 2011
Igarashi 2023
Khan 2023
Shoemaker 2012
Tager 2014
Benaim 2019
Claeys 2024
Kwok 2013
Kaleth 2016
Gardner 2018
Fulk 2018
Lika 2024
Mostafaee2023
King 2022
Oosterveer 2022
Spina 2019
Zeitleberger 2021
Antonescu 2014
Sheraz 2022
Anchor
Distribution
Opinion Delphi
closed shape= exercise intervention
open shape= no intervention
half filled shape= medical intervention

Figure S2 Calculated Minimal Important Difference for the Incremental Shuttle Walking Test for all included studies.

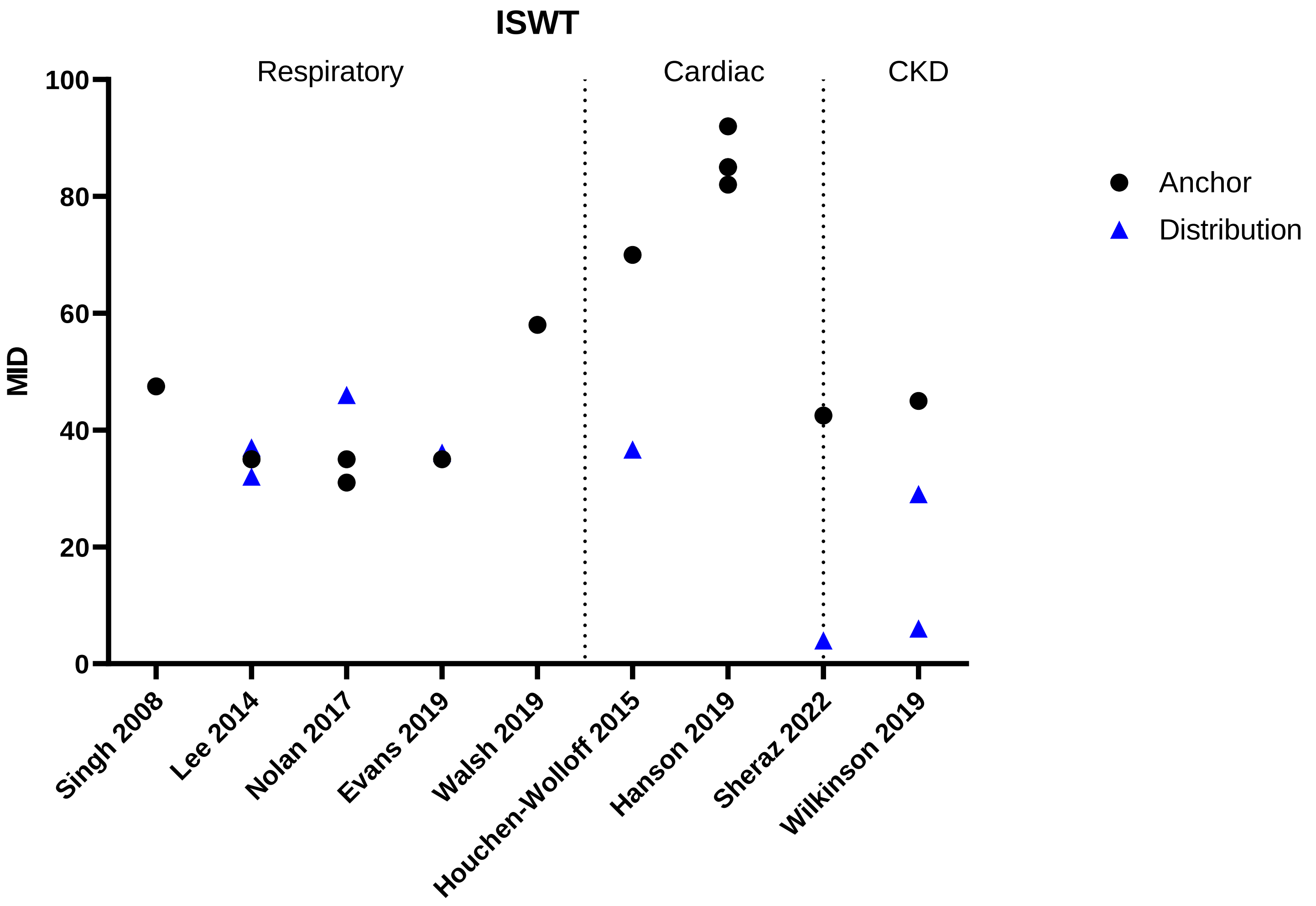

Figure S3 Calcuted Minimal Important Difference for the Endurance Shuttle Walking Test for all included studies

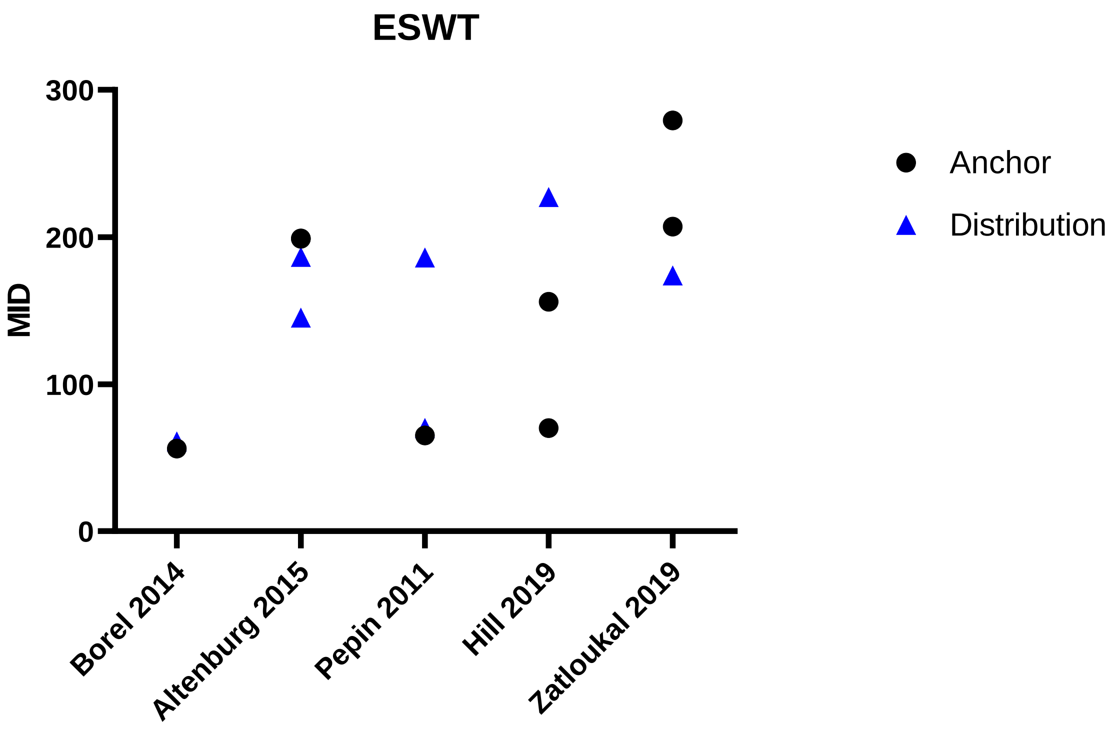

Table s1 Calculated Minimal Important Differences.

Test	Method of generating MID	N=reports (patients)	Calculated MID
**6MWD (m)**			
All	Anchor- GRCQ	**6(336)**	**27(23, 31)**
	Anchor- all	**8(1378)**	**26(22, 30)**
	Distribution SEM	**13(3011)**	**13-45**
	Effect size	**5(1074)**	**20-42**
Respiratory	Anchor- GRCQ	**3 (160)**	**27(21, 34)**
	Anchor- all	**5 (1415)**	**25(24, 26)**
	Distribution SEM	**6(2087)**	**22-45**
	Effect size	**4(1032)**	**20-42**
Cardiac	Anchor- GRCQ	**2 (103)**	**23(8, 37)**
	Anchor- all	**2 (103)**	**23(8, 37)**
	Distribution SEM	**3 (631)**	**13-37**
	Effect size	**0**	**n/a**
MSK/Neuro	Anchor- GRCQ	**1 (73)**	**37(26, 49)**
	Anchor-all	**1 (73)**	**37(26, 49)**
	Distribution SEM	**4 (732)**	**13-58**
	Effect size	**1 (42)**	**20**
Renal	Anchor- GRCQ	**0**	**No data**
	Anchor-all	**0**	**No data**
	Distribution SEM	**0**	**No data**
	Effect size	**0**	**No data**
**ISWT (m)**			
All	Anchor- GRCQ	**6(1366)**	**52(18, 86)**
	Anchor- all	**8(1511)**	**53(43, 62)**
	Distribution SEM	**6(444)**	**4-70**
	Effect size	**3(722)**	**32-70**
Respiratory	Anchor- GRCQ	**4 (1094)**	**45(37,52)**
	Anchor- all	**5 (1213)**	**47(39, 57)**
	Distribution SEM	**2 (109)**	**31-37**
	Effect size	**3 (722)**	**32-36**
Cardiac	Anchor- GRCQ	**2 (272)**	**70 (55, 85)**
	Anchor- all	**2 (272)**	**70(55, 85)**
	Distribution SEM	**2 (309)**	**4-37**
	Effect size	**0**	**n/a**
MSK/Neuro	Anchor- GRCQ	**0**	**No data**
	Anchor- all	**0**	**No data**
	Distribution SEM	**0**	**No data**
	Effect size	**0**	**No data**
Renal	Anchor- GRCQ	**1(26)**	**45[21]**
	Anchor- all	**1**	**45[21]**
	Distribution SEM	**1(26)**	**6**
	Effect size	**0**	**No data**
**ESWT (seconds)**			
All	Anchor- GRCQ	**5 (1141)**	**123(36, 209)**
	Anchor- all	**8 (1141)**	**159(94, 224)**
	Distribution SEM	**2(354)**	**144 (61, 227)**
	Effect size	**0**	**No data**
Respiratory	Anchor- GRCQ	**5 (1141)**	**123(36, 209)**
	Anchor- all	**8 (1141)**	**159(94, 224)**
	Distribution SEM	**2(354)**	**144 (61, 227)**
	Effect size	**0**	**No data**
Cardiac	Anchor- GRCQ	**0**	**No data**
	Anchor- all	**0**	**No data**
	Distribution SEM	**0**	**No data**
	Effect size	**0**	**No data**
MSK/Neuro	Anchor- GRCQ	**0**	**No data**
	Anchor- all	**0**	**No data**
	Distribution SEM	**0**	**No data**
	Effect size	**0**	**No data**
Renal	Anchor- GRCQ	**0**	**No data**
	Anchor- all	**0**	**No data**
	Distribution SEM	**0**	**No data**
	Effect size	**0**	**No data**

Table s1 Calculated Minimal Important Difference (MID) per disease group and methodology. Presented as mean[SD] or mean(CI) 6MWD 6 Minute Walk Distance, ISWT Incremental Shuttle Walking Test, ESWT

Endurance Shuttle Walking Test, SEM Standard Error of Measurement, SD Standard Deviation, GRCQ Global Rating of Change Questionnaire, MSK Musculoskeletal *not able to calculate as range not available.
